# Effect of autologous platelet concentrates on osteoblast cell activity during early osseointegration

**DOI:** 10.6026/973206300210441

**Published:** 2025-03-31

**Authors:** Mohan Raj J.P, Aarani P., Johnson Raja James, Jacob Raja, Divya S., Thamilselvan M.

**Affiliations:** 1 Department of Periodontology, Rajas Dental College and Hospital, Tirunelveli, Tamil Nadu, India; 2 Department of Prosthodontist, Private Practitioner, Tamil Nadu, India

**Keywords:** Osseointegration, autologous platelet concentrates, platelet rich fibrin, growth factors, biomaterials in implantology

## Abstract

Advances in implant dentistry focuses on enhancing the predictability of implant success using advanced regenerative techniques. Autologous platelet concentrates rich in growth factors play a crucial role in stimulating osteoblast activity and improving osseointegration at implant sites. Therefore, it is of interest to evaluate the osteogenic efficacy of four injectable platelet concentrates: (1) injectable-platelet-rich fibrin, (2) leukocyte platelet-rich fibrin, (3) advanced platelet-rich fibrin and (4) Advanced platelet-rich fibrin + on titanium surfaces. All tested concentrates enhanced osteoblastic adhesion, differentiation and mineralization, with injectable-platelet-rich fibrin demonstrating the most significant calcium nodule formation. Thus, injectable-platelet-rich fibrin proved to be the most effective in promoting early osseointegration compared to the other platelet-rich fibrin types.

## Background:

Dental Implantology is an advanced treatment modality for the rehabilitation of missing dentition. Increasing the predictability of implant success rates is a global research focus. Autologous platelet concentrates, which serve as a rich source of growth factors, enhance osseointegration around the implant site by promoting osteoblast cell activity. To enhance the predictability of implant placement, research has focused on various types of injectable autologous platelet concentrates [1]. Platelet-Rich Fibrin, the second generation of platelet concentrate has been defined as an autologous platelet and leukocyte-rich fibrin biomaterial, which intends to accumulate platelets, immunity promoters and released cytokines in the fibrin clot. Some of the most important growth factors of Platelet-Rich Fibrin include transforming growth factor β, platelet-derived growth factor, insulin-like growth factor 1, vascular endothelial growth factor and epidermal growth factor [2]. Platelet-Rich Fibrin, prepared without any anticoagulants or additives, has the potential to accelerate healing without negatively impacting the coagulation cascade. It is also known for advantages such as less handling time, slow sustained release of growth factors and better entrapment of leukocytes due to its fibrin mesh structure. Initially, Platelet-Rich Fibrin was used at high centrifugal speeds, utilizing the fibrin clot as a three-dimensional scaffold. However, liquid Platelet-Rich Fibrin has garnered interest among clinicians due to its cell composition and fibrin content. Liquid injectable concentrates can be obtained by slow fibrin coagulation at early time points using non-glass centrifugation tubes [3].

Over the past decade, efforts have been made to develop injectable autologous platelet concentrates by altering the speed and centrifugation time. Injectable autologous platelet concentrates are now extending their regenerative potential to improve the predictability of implant treatment outcomes by enhancing osseointegration around dental implant sites [4- 5]. The aim of this study is to evaluate and compare the individual osteogenic responses of four types of Platelet-Rich Fibrin concentrates-injectable Platelet-Rich Fibrin (i-PRF), Leukocyte Platelet-Rich Fibrin (L-PRF), Advanced Platelet-Rich Fibrin and Advanced Platelet-Rich Fibrin Plus (Advanced platelet-rich fibrin +)-in terms of their efficiency in regulating osteoblastic differentiation and mineralization on titanium surfaces.

## Materials and Methods:

## Study design:

This was an in vitro experimental study conducted using the MG63 cell line cultured on titanium discs. These discs were cultured with the incorporation of four types of platelet-rich fibrin. A titanium disc with no platelet-rich fibrin was used as the control group. To compare the effects of the different injectable platelet concentrates, cell viability and mineralized nodule assays were performed.

## Osteoblast cell culture:

Commercially available MG63 cell lines were used for the *in-vitro* culture on titanium discs [4, 5]. This osteoblastic cell line was obtained from IBMS, Taramani Campus and Chennai, India. MG63 cells exhibit high alkaline phosphatase activity and osteocalcin production [6], making those comparable to human osteoblasts as they are derived from human osteosarcoma [7, 8]. The Dulbecco's Modified Eagle's Medium was placed in a humidified incubator (5% CO2 at 37 ± 0.2°C) to promote cell growth. The culture medium also included 10% Fetal Bovine Serum containing antibiotics-penicillin, streptomycin and amphotericin B (5000 units) [9, 10, 11 and 12]. A phase-contrast inverted light microscope was used for regular monitoring and the culture medium was changed every three days.

## Preparation of platelet-rich fibrin:

Four groups were prepared from a single healthy male volunteer with informed consent by collecting 10 ml of whole blood without anticoagulants and subjecting them to varying centrifugal (Figure 1 - see PDF) processes.

## Preparation of grade 5 titanium discs:

Grade 5 Titanium alloy (Ti6Al4V) (Implant Biomaterial-Noble Biocare USA) discs were used. This alloy has been used to produce osseointegration in both *in-vitro* [13, 14] and *in-vivo* studies [15, 16]. The osteoblastic cell line was seeded (20% conditioned media at a density of 10,000 cells) on discs with a thickness of 1 mm and a diameter of 5 mm (Figure 2a - see PDF & Figure 2b - see PDF). The titanium alloy discs seeded with osteoblastic cells were cultured with and without platelet rich fibrin. The study groups (Group A: injectable Platelet-Rich Fibrin, Group B: leukocyte platelet-rich fibrin, Group C: Advanced platelet-rich fibrin, Group D: Advanced platelet-rich fibrin + and control group (Group E: Control) were obtained (Figure 3 - see PDF).

## Assays conducted:

The following assays were performed on each sample immediately after a 30-minute exposure to UV irradiation - MTT Assay, Mineralized nodule assay, Alizarin red staining.

## MTT Assay (after 24 hours):

Used for evaluating cell viability, this assay quantifies the percentage of surviving fibroblast cells by monitoring changes in cell morphology through a phase contrast microscope. The mitochondrial enzyme succinate dehydrogenase activity, which reduces tetrazolium salts to formazan crystals, helps to evaluate cell viability. The colour intensity is directly related to the viable cell count.

## Mineralized nodule assay:

Alkaline Phosphatase Activity Test: Conducted on days 1, 3 and 7 after seeding, this test quantifies Alkaline Phosphatase activity in cells seeded on 2D scaffolds using spectrophotometric evaluation at 405 nm.

## Alizarin red staining:

After sufficient growth of the samples, advanced platelet-rich fibrin, advanced platelet-rich fibrin +, leukocyte platelet-rich fibrin and injectable-platelet-rich fibrin were added and incubated. Subcultures from each group were stained with Alizarin Red after 14 days to analyze calcium nodules using a phase contrast microscope. Quantitative analysis was performed by determining OD 405 values of known Alizarin Red concentrations and comparing them with those from unknown samples.

## Statistical analysis:

Statistical analysis was conducted using SPSS 22.0 software. Results were depicted in terms of mean ± standard deviation. One-way ANOVA was performed for inter-group comparison and post-hoc analysis was done for intra-group comparison.

## Results:

## Cell viability assay:

The formula used for calculating cell viability is:

Cell Viability % = OD (Test Sample) - OD (Blank) / OD (Control) - OD (Blank) x 100

The viability of different platelet-rich fibrin variants varies due to differences in their preparation and release profiles. Advanced platelet-rich fibrin + exhibited the highest viability at 93%, attributed to its lower centrifugation speeds and modified protocols that preserve more cellular components and create a denser fibrin matrix. This allows for a more controlled and prolonged release of growth factors, supporting sustained cell proliferation and survival. Advanced platelet-rich fibrin follows with 89% viability, benefiting from lower centrifugation speeds and moderate growth factor release, although it is slightly less enriched compared to advanced platelet-rich fibrin. Leukocyte platelet-rich fibrin, with 85% viability, is more solid and less porous, which can slow the release of growth factors and contribute to slightly lower viability. In contrast, injectable Platelet-Rich Fibrin, known for its rapid release profile, shows the lowest viability, which may be due to the lack of a long-term supply of growth factors that affects cell survival (Table 1).

## Mineralized nodule assay:

Alkaline Phosphatase plays a crucial role as an early marker of osteoblast differentiation. In this study, alkaline phosphatase activity in cells seeded on 2D scaffolds was quantified spectro-photometrically at 405 nm. As shown in Table 2, the control group exhibited the lowest alkaline phosphatase activity (4.131), indicating minimal osteogenic potential and highlighting the need for biological enhancement to promote osseointegration. Among the platelet-rich fibrin types, injectable-platelet-rich fibrin (26.9892) demonstrated the highest osteogenic response, suggesting superior osteoblast stimulation, mineralization, and early bone integration. Advanced platelet-rich fibrin + (23.0882) followed closely, showing strong regenerative potential but slightly lower efficacy than injectable Platelet-Rich Fibrin. Advanced platelet-rich fibrin (16.9892) exhibited moderate osteogenic activity, making it a viable but less potent option. Leukocyte platelet-rich fibrin (9.4786) showed the lowest response among platelet-rich fibrin variants, though it still significantly outperformed the control, indicating some osteogenic potential. These findings are further illustrated in Figure 5 (see PDF), where injectable-platelet-rich fibrin demonstrated the highest alkaline phosphatase activity after 14 days, a statistically significant result. Conversely, leukocyte platelet-rich fibrin exhibited the lowest alkaline phosphatase activity. The osteoblast population concentration was highest in the injectable-platelet-rich fibrin group, followed by advanced platelet-rich fibrin +, Advanced platelet-rich fibrin, and leukocyte platelet-rich fibrin, with the control group showing the least. The results are presented in both tabular (Table 2) and graphical (Figure 5 - see PDF) formats for clarity.

The phase contrast resolution of mineralized nodules (Figure 4 - see PDF) reveals the mean concentration of osteoblast populations in both test and control groups, as documented in Table 3. The control group shows the lowest osteoblast concentration (29.54 µg), indicating minimal osteogenic potential. Among the platelet-rich fibrin groups, injectable-platelet-rich fibrin (53.65 µg) exhibits the highest osteoblast concentration, suggesting superior regenerative potential. Advanced platelet-rich fibrin + (52.43 µg) follow closely, demonstrating strong osteogenic activity. Advanced platelet-rich fibrin (48.24 µg) and leukocyte platelet-rich fibrin (46.00 µg) also show significant osteoblast concentrations, though slightly lower than those observed with injectable-platelet-rich fibrin and advanced platelet-rich fibrin +. The results are presented in Figure 6 - (see PDF).

Results of ANOVA for the cell viability assay showed statistical significance on inter-group comparison (Table 4). Intra-group comparisons for alkaline phosphatase and alizarin red staining among various study groups also showed statistically significant results (p < 0.001) (Table 5, Table 6).

## Discussion:

The aim of this study was to evaluate the efficacy of growth factors in accelerating the osteogenic response in osteoblast cells and to compare and evaluate the individual osteogenic responses of four different injectable autologous platelet concentrates: injectable Platelet-Rich Fibrin, leukocyte platelet-rich fibrin, advanced platelet-rich fibrin and advanced platelet-rich fibrin + in terms of osteoblastic differentiation and mineralization on the surface of titanium disks. Over a decade after the development of autologous platelet concentrates by Choukroun in 2001, membranous and liquid versions of Platelet-Rich Fibrin (PRF) have evolved, [6, 7-8] with many clinicians being fascinated by the handling properties of injectable-platelet-rich fibrin.

Various studies compared the autologous platelet concentrates and it was found that they accelerated osteogenic healing process, on comparing various implant surfaces by coating it with PRP and leukocyte platelet-rich fibrin results were found that leukocyte platelet-rich fibrin promoted better implant stability by reducing the marginal bone loss and early wound healing. But although they had better osteogenic activity and early wound healing further data is required on its influence on the bone formation progress. Liza *et al.*. in their study compared injectable-platelet rich fibrin, advanced platelet rich fibrin and enamel matrix derivatives on contaminated Titanium disc the results showed positive increase in osteoblast activity, on considering the other factors injectable Platelet-Rich Fibrin was found to promote re-osseointegration and tissue healing [9- 10]. This study sheds light on the efficacy of four different injectable Platelet-Rich Fibrin types during the early osseointegration period. Correlating the observations of our study with evidence from other scholars, it is evident that the addition of autologous platelet derivatives positively influences osteogenic responses on titanium surfaces. Among these, injectable-platelet-rich fibrin, followed by advanced platelet-rich fibrin +, has shown a significantly remarkable influence in promoting osteogenic activity [11]. Injectable-platelet-rich fibrin presents higher concentrations of regenerative cells and growth factors compared to its conventional counterpart as a result of reduced centrifugation speed. Additionally, there is a difference in cytokine content between injectable-platelet-rich fibrin and leukocyte platelet-rich fibrin, with the former being enriched with interleukin 10 (IL-10), a cytokine involved in reducing inflammatory mediators and prompting tissue regeneration. This liquid formulation of platelet concentrate offers successful clinical applicability for the clinicians to readily apply the biomaterial alone or in combination with other biomaterials in order to promote bone regeneration [12]. In the case of Advanced platelet-rich fibrin +, the underlying contributing factor to osteogenic activity is hypothesized to be the shorter centrifugation duration. Recent studies have suggested that decreasing centrifugation speeds can maintain a higher proportion of leukocytes in the upper layer of Platelet-Rich Fibrin, thereby increasing total growth factor release [13, 14, 15, 16, 17-18].

## Conclusion:

All the autologous growth factors positively influenced osseointegration with injectable-platelet-rich fibrin showing a better influence. Advanced platelet-rich fibrin also demonstrated higher values than the others, though the difference was not remarkably significant. Therefore, all autologous growth factors improve proliferation and differentiation activity, which plays an important role in modulating osseointegration of implants. Enhanced early osseointegration of implants would provide predictable osseointegration and earlier functional loading. Further studies are required to corroborate and reinforce these findings.

## Declaration of interest statement:

I, Dr Mohan Raj JP, declare that there are no conflicts of interest related to the research study and I confirm that I have no financial or personal relationships with other people or organizations that could inappropriately influence my work. All sources of funding for this study have been Self-funded.

## Figures and Tables

**Table 1:** MTT assay of test and control group

**Group A: i-PRF**	**Group B: L-PRF**	**Group C: A-PRF**	**Group D:APRF+**	**Control Group**
82	85	89	93	60

**Table 2:** Alkaline phosphatase activity between test and control group

**Group A: i-PRF**	**Group B: L-PRF**	**Group C: A-PRF**	**Group D:APRF+**	**Control Group**
26.9892	9.4786	16.9892	23.0882	4.131

**Table 3:** Concentration of osteoblast population of test and control group

**Group A: i-PRF**	**Group B: L-PRF**	**Group C: A-PRF**	**Group D:APRF+**	**Control Group**
53.65	46	48.24	52.43	29.54

**Table 4:** Results of inter group analysis of variance (ANOVA)

**Alkaline phosphatase**	**Group A**	**24**	**26.9892**	**0.6073**	
	Group B	24	9.4786	0.4199	
	Group C	24	16.9892	0.4095	< 0.001*
	Group D	24	23.0882	0.2694	
	Group E	24	4.131	0.2133	
	Total	120	16.1352	8.4744	
alizarin red staining	Group A	24	53.65	0.5354	
	Group B	24	46	0.1574	
	Group C	24	48.24	0.1619	< 0.001*
	Group D	24	52.43	0.34	
	Group E	24	29.54	0.4132	
	Total	120	45.972	8.7129	

**Table 5:** Results of for multiple comparisons using bonferroni correction (Post-hoc Analysis) for alkaline phosphatase

	**Groups**	**Groups**	**Mean Difference**	**p-value**
	Group A	Group B	17.5106	< 0.001*
		Group C	10	< 0.001*
		Group D	3.901	< 0.001*
		Group E	22.8582	< 0.001*
Alkaline phosphatase	Group B	Group C	7.5106	< 0.001*
		Group D	13.6096	< 0.001*
		Group E	5.3476	< 0.001*
	Group C	Group D	6.099	< 0.001*
		Group E	12.8582	< 0.001*
	Group D	Group E	18.9572	< 0.001*
P-value based on Analysis of Variance (ANOVA)
after adjusting for multiple comparisons using Bonferroni Correction
(Post-hoc Analysis)* = Statistically Significant (p < 0.05)

**Table 6:** Results of for multiple comparisons using Bonferroni Correction (Post-hoc Analysis) for ARS (alizarin red staining)

	**Groups**	**Groups**	**Mean Difference**	**p-value**
	Group A	Group B	7.65	< 0.001*
		Group C	5.41	< 0.001*
		Group D	1.22	< 0.001*
		Group E	24.11	< 0.001*
ARS	Group B	Group C	2.24	< 0.001*
		Group D	6.43	< 0.001*
		Group E	16.46	< 0.001*
	Group C	Group D	4.19	< 0.001*
		Group E	18.7	< 0.001*
	Group D	Group E	22.89	< 0.001*
P-value based on Analysis of Variance (ANOVA) after
adjusting for multiple comparisons using Bonferroni Correction
(Post-hoc Analysis)* = Statistically Significant (p < 0.05)
